# Supplementary material for: Visualization of oxygen distribution patterns caused by coral and algae
Source: PeerJ. 2013 Jul 16;1:e106. doi: 10.7717/peerj.106 (PMC3719126; doi:10.7717/peerj.106)
Supplement: Supplemental Information 1 — Matlab script to generate colormap images of spatial oxygen dynamics. [file peerj-01-106-s001.docx]

R=imread('red channel image','TIF');

G=imread('green channel image','TIF');

rg=R-G;

rrg=double(rg)./double(G);

imtool(rrg)

R0=2.33;

KSV=.018659;

a=3.229E-13;

o2=(R0-rrg)./(KSV*(rrg-R0*a));

O2=(o2>700)*700+(o2<0)*0+((o2>0)&(o2<700)).*o2;

flipped_O2=flipud(O2);

pcolor(flipped_O2)

shading interp

shg
