# Supplementary figures and images for: Visualization of oxygen distribution patterns caused by coral and algae
Source: PeerJ. 2013 Jul 16;1:e106. doi: 10.7717/peerj.106 (PMC3719126; doi:10.7717/peerj.106)

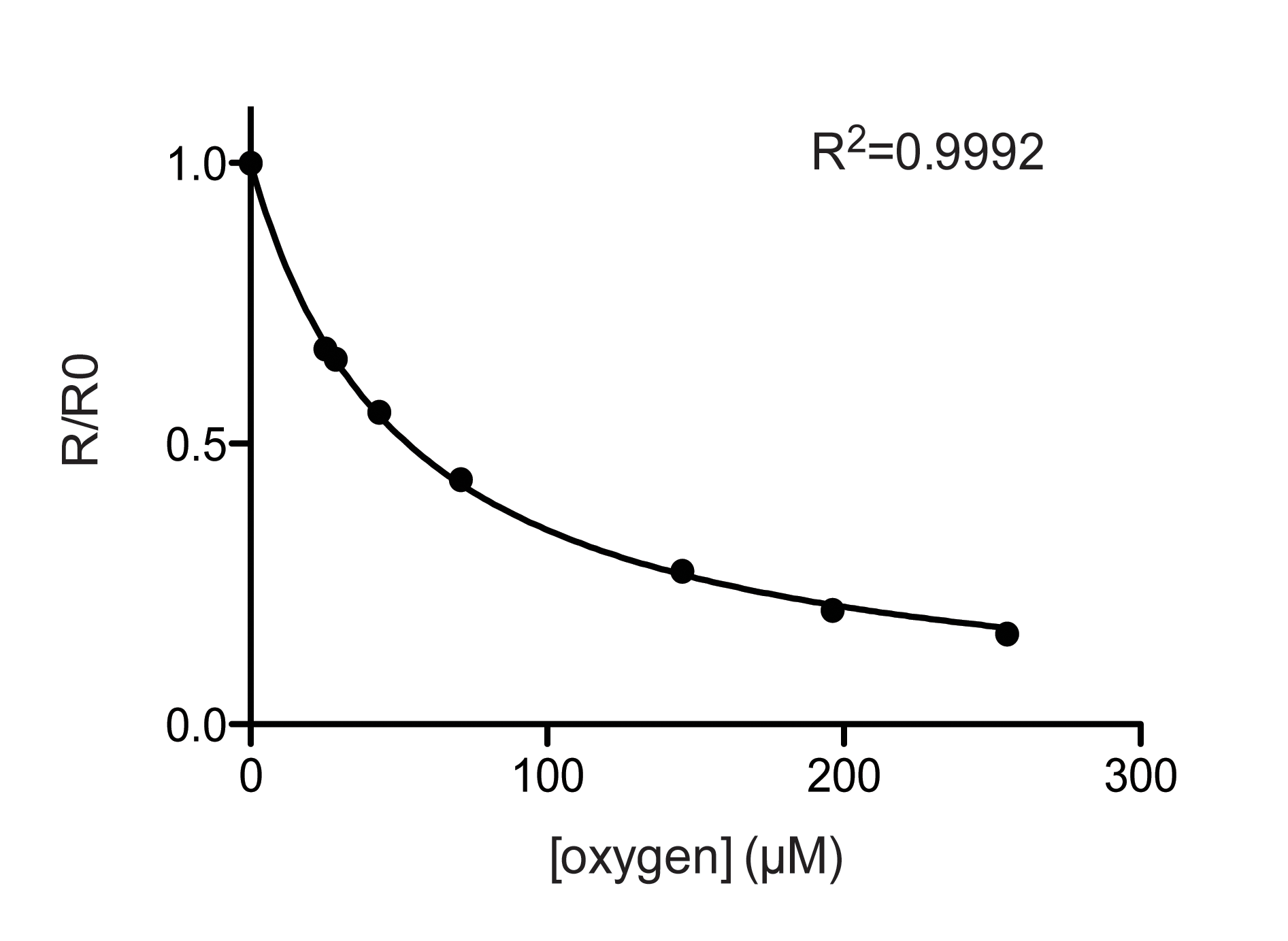

Supplement: Figure S1 — Optode calibration was done with a non-linear regression model using the one site Stern-Volmer equation (Eq. (2)). Stern-Volmer constant (Ksv = 6.1 × 10^−3^ µM−1) and unquenched portion of the indicator (1.27 × 10^−9^) for the here used optode sheet was derived from this regression. [file peerj-01-106-s002.png]
